# CRISPR-like sequences in *Helicobacter pylori* and application in genotyping

**DOI:** 10.1186/s13099-017-0215-8

**Published:** 2017-11-17

**Authors:** Khotchawan Bangpanwimon, Jaksin Sottisuporn, Pimonsri Mittraparp-arthorn, Warattaya Ueaphatthanaphanich, Attapon Rattanasupar, Christine Pourcel, Varaporn Vuddhakul

**Affiliations:** 1 Department of Microbiology, Faculty of Science, Prince of Songkla University, Hat Yai, Thailand; 2 NKC Institute of Gastroenterology and Hepatology, Songklanagarind Hospital, Faculty of Medicine, Prince of Songkla University, Hat Yai, Thailand; 3 Microbiology Laboratory, Vichaiyut Hospital, Bangkok, Thailand; 4 KC Center of Gastroenterology and Hepatology, Hat Yai Hospital, Hat Yai, Thailand; 5 Institute for Integrative Biology of the Cell (I2BC), CEA, CNRS, Univ. Paris-Sud, Université Paris-Saclay, Gif-sur-Yvette, France

**Keywords:** *Helicobacter pylori*, *vacA*-*like* gene, *vlpC* gene, Orphan CRISPR array, CRISPR-like sequences, CRISPR-virulence typing

## Abstract

**Background:**

Many bacteria and archaea possess a defense system called clustered regularly interspaced short palindromic repeats (CRISPR) associated proteins (CRISPR-Cas system) against invaders such as phages or plasmids. This system has not been demonstrated in *Helicobacter pylori*. The numbers of spacer in CRISPR array differ among bacterial strains and can be used as a genetic marker for bacterial typing.

**Results:**

A total of 36 *H. pylori* isolates were collected from patients in three hospitals located in the central (PBH) and southern (SKH) regions of Thailand. It is of interest that CRISPR-like sequences of this bacterium were detected in *vlpC* encoded for VacA-like protein C. Virulence genes were investigated and the most pathogenic genotype (*cagA vacA* s1m1) was detected in 17 out of 29 (58.6%) isolates from PBH and 5 out of 7 (71.4%) from SKH. *vapD* gene was identified in each one isolate from PBH and SKH. CRISPR-like sequences and virulence genes of 20 isolates of *H. pylori* obtained in this study were analyzed and CRISPR-virulence typing was constructed and compared to profiles obtained by the random amplification of polymorphic DNA (RAPD) technique. The discriminatory power (DI) of CRISPR-virulence typing was not different from RAPD typing.

**Conclusion:**

CRISPR-virulence typing in *H. pylori* is easy and reliable for epidemiology and can be used for inter-laboratory interpretation.

**Electronic supplementary material:**

The online version of this article (10.1186/s13099-017-0215-8) contains supplementary material, which is available to authorized users.

## Background

Clustered regularly interspaced short palindromic repeats (CRISPR) are detected in around 40% of bacteria and many archaea [1, 2]. CRISPR together with the CRISPR-associated genes (*cas*) are a prokaryotic defense system against invasive bacteriophages or genetic elements. Cas proteins function to degrade foreign nucleic acids of bacteriophages or plasmids. The number of CRISPR loci detected vary between and within bacterial species and strains, 1–4 in *Escherichia coli* [3], 3 in *Yersinia pestis* [4], 2 in *Salmonella* Typhimurium [5], and 1–2 in *Staphylococcus aureus* [6]. CRISPR loci contain multiple direct repeat (DRs) sequences from 21 to 48 bp long separated by variable spacer sequences 21–72 bp in length [7]. DR sequences are commonly conserved whereas spacer sequences are diverse, and derived from bacteriophages or plasmids. The variable number of DRs and spacers have been used as a typing tool in epidemiologic and evolutionary analysis of bacterial strains [8].

*Helicobacter pylori* is a Gram negative bacterium that causes peptic ulcer, gastric cancer and mucosa associated lymphoid tissue lymphoma. The risk of disease is associated with *H. pylori* harboring the cytotoxin associated gene A and vacuolating cytotoxin A, encoded by *cagA* and *vacA* genes respectively. The *cagA* gene encodes the bacterial oncoprotein that causes abnormal cellular signals leading to deregulation of cell growth, cell turnover, cell to cell contact, and elongation of epithelial cells. The *vacA* gene encodes the pore-forming toxin that induces epithelial cell apoptosis, and inhibits leukocyte activation by massive vacuolization, and disruption of the endosome [9]. Allelic variation of *vacA* occurs in a signal region (s1/s2) and a middle region (m1/m2) resulting in different levels of vacuolating cytotoxicity. The vacuolating activity is high in the s1m1 genotype whereas the intermediate and absent activities are associated with s1m2 and s2m2 genotypes, respectively. *H. pylori* carrying the *cagA* and *vacA* s1m1 allele has been frequently isolated from patients with severe gastric diseases including peptic ulcers, atrophic gastritis, and gastric cancer [10]. The gene encoded for the virulence-associated protein D (*vapD*) is present in all virulent strains of ovine footrot bacteria, *Dichelobacter nodosus*, although the VapD protein function in this bacterium is unknown [11]. This gene has been reported in some *H. pylori* isolates with 64.9% nucleotide identity to the *vapD* gene of *D. nodosus* [12]. The *vap* region of *D. nodosus* has been demonstrated to harbor genetic element of bacteriophage suggesting the possibility that gene in this region may be transferred among bacteria [13].

In studies of *H. pylori*, biotyping, serotyping and hemagglutinin typing, have been reported to possess low discriminatory power index (DI) compared to genotyping such as RAPD [14], pulse field gel electrophoresis (PFGE) [15], restriction fragment length polymorphism-PCR (RFLP-PCR) [16], and repetitive extragenic palindromic PCR fingerprinting (REP-PCR) [17]. PFGE is not widely used for *H. pylori* because inter-patient variation is rare in the fingerprints obtained [18]. The DI of PFGE is between 0.24 and 0.88 whereas RAPD analysis reveals excellent DI (between 0.99 and 1). Thus, RAPD is recommended for *H. pylori* typing [19].

Analysis of the CRISPR-Cas systems in *H. pylori* has not been clearly demonstrated. The polymorphism detected in CRISPR loci has been applied as a genetic marker for typing many bacteria, such as *Campylobacter fetus* [20] and *S. *Typhimurium [5]. In the present study, the CRISPR sequences and virulence genes of this bacterium were investigated and CRISPR-virulence typing was compared to the RAPD.

## Methods

### Isolation and identification of *H. pylori*

Gastric biopsy samples were obtained from the antral area of patient stomachs in a private hospital in Bangkok (PBH), and two government hospitals in Songkhla (SKH), in the central and southern regions of Thailand, respectively. They were transported in 0.5 ml BHI broth with vancomycin 10 μg/ml, amphotericin B 10 μg/ml, trimethoprim 5 μg/ml and polymyxin B 2500 IU/l. The samples were cultured on Columbia sheep blood agar (CBA) and incubated at 37 °C for 3–7 days under microaerophilic conditions (Oxoid, CampyGen™ gas generator, United Kingdom). The identification of *H. pylori* was based on colony morphology and biochemical testing. Confirmation was performed by PCR targeted to the *ureC* (*glmM*) gene using forward primer (5′-AAGCTTTTAGGGGTGTTAGGGGTTT-3′) and reverse primer (5′-AAGCTTACTTTCTAACACTAACGC-3′) to detect a 294 bp gene fragment [21].

### Investigation of CRISPR region in *H. pylori*

Five whole genomes of *H. pylori* strains (26695-1MET, XZ274, F57, India7, and SNT49) were analyzed for CRISPR loci using the CRISPRfinder server [22], and specific primers were designed (Table 1). PCR was carried out using PCR mixture containing 5× PrimeSTAR GXL buffer (Mg2+ plus), 2.5 U PrimeSTAR GXL DNA high-fidelity polymerase (Takara, Shiga, Japan), 0.3 mM dNTPs, 0.4 μM of forward and reverse CRISPR-HP primers, and 10 μl of template DNA in a total volume of 100 μl. The PCR process included initial denaturation at 95 °C for 5 min, followed by 35 cycles of denaturation at 95 °C for 1 min, annealing at 56 °C for 1 min, and extension at 68 °C for 1 min with a final extension at 68 °C for 10 min. The PCR products were purified and sequenced.

**Table 1:** Primers used for detection of virulence genes and CRISPR locus of *H. pylori*

DNA region(s) amplified	Primer name	Primer sequence	Amplicon sizes(s) (bp)	References or sources
*vacA* s1/s2	VAI-F VAI-R	5′-ATGGAAATACAACAAACACAC-3′ 5′-CTGCTTGAATGCGCCAAAC-3′	259/286	[24]
*vacA* m1/m2	VAG-F VAG-R	5′-CAATCTGTCCAATCAAGCGAG-3′ 5′-GCGTCAAAATAATTCCAAGG-3′	567/642	[24]
*cagA*	Cag5c-F Cag5c-R	5′-GTTGATAACGCTGTCGCTTC-3′ 5′-GGGTTGTATGATATTTTCCATAA-3′	350	[24]
*vapD*	D1-F D2-R	5′-AGAGATGCGGTGAATGG-3′ 5′-AAGCGTTATGAGTGGTGTG-3′	498	[25]
CRISPR locus	CRISPR-HP-F CRISPR-HP-R	5′-ATGGGGGCTTTAGTTTCAG-3′ 5′-TAGCAAAAGGCGAACTTGA-3′	Variable	This study

The direct repeats (DRs) and spacers of the CRISPR loci were analyzed using the CRISPRfinder server. DRs were grouped based on the similarity of consensus direct repeat sequences (CDRs) of each isolate. Clusters of DRs were assigned by multiple sequence alignment (MSA) using MEGA7 software [23]. The spacers of CRISPR were classified according to a phylogenetic tree inferred using MEGA7 software, and the spacer sequences were analyzed using the CRISPRTarget tool (http://bioanalysis.otago.ac.nz/CRISPRTarget/).

### Virulence genes investigation

Multiplex PCR was performed to detect *H. pylori* toxin genes, the *cagA* gene, and the s and m regions of the *vacA* gene [24]. DNA template was prepared by boiling technique. The *vapD* gene was investigated by single PCR, as previously described [25].

### RAPD genotyping

RAPD genotyping was carried out as described previously using primer 1281 (5′-AACGCGCAAC-3′). The RAPD profiles were analyzed with BioNumerics 7.6 (Applied Maths, Belgium), and a dendrogram was constructed by UPGMA method using the Dice similarity coefficient [26].

### CRISPR-virulence typing

CRISPR-virulence typing was based on the CRISPR spacer sequences and the presence of *cagA*, and *vacA* s and m regions. A profile of each isolate was created using a binary matrix of presence or absence of spacer sequences and virulence genes. The dendrogram was constructed using BioNumerics 7.6 software with the UPGMA algorithm using the Dice similarity coefficient. The DI of putative CRISPR-virulence typing and RAPD typing were assessed by Simpson’s diversity index [27].

## Results

### Isolation of *H. pylori* and identification of CRISPR-like sequences

A total of 353 gastric biopsy samples were collected from PBH and 80 from SKH. Twenty-nine (8.2%) and seven (8.7%) isolates of *H. pylori* were identified from PBH and SKH, respectively. Twenty isolates of *H. pylori* (13 from PBH and 7 from SKH) were selected for CRISPR analysis and the CRISPR-like sequences were detected in all of the isolates by PCR with amplification products of 280–380 bp. Using the CRISPRfinder server, the numbers of DRs were between 2 and 4 with lengths of 23–31 bp (Table 2). They were divided into 13 DR groups and classified into four clusters (DR-A, B, C and D), each cluster shared specific sequences within the cluster (Additional file 1: Figure S1). The conserved sequences of DRs of all *H. pylori* isolates were TTCAATCAAGG(G/C)ACTTA (Table 2).

**Table 2:** Characteristics of CRISPR-like loci in 20 *H. pylori* isolates

Isolate	Consensus direct repeats (CDRs) sequences	No. of DRs	No. of spacers	CRISPR-like locus pattern (bp)
PBH01	AACAGCACTTTCAATCAAGGGACTTACAA	4	3	*29*-*31*-*29*-*28*-*29*-*31*-*29*
PBH02	TTCAATCAAGGGACTTATAAATT	3	2	*23*-*37*-*23*-*31*-*23*
PBH03	TTCAATCAAGGGACTTATAGCTT	3	2	*23*-*37*-*23*-*37*-*23*
PBH04	TTCAATCAAGGGACTTATAAATT	4	3	*23*-*37*-*23*-*34*-*23*-*31*-*23*
PBH05	TTCAATCAAGGGACTTATAATTTTA	4	3	*25*-*35*-*25*-*32*-*25*-*29*-*25*
PBH06	TTCAATCAAGGGACTTACAAATT	4	3	*23*-37-*23*-*34*-*23*-*37*-*23*
PBH07	CACTTTCAATCAAGGGACTTACAACTTTAAT	3	2	*31*-*29*-*31*-*26*-*31*
PBH08	TTCAATCAAGGGACTTACAAATT	4	3	*23*-*37*-*23*-*34*-*23*-*37*-*23*
PBH09	TTCAATCAAGGGACTTATAAATT	3	2	*23*-*37*-*23*-*31*-*23*
PBH10	TTCAATCAAGGGACTTATGACTTTAAT	2	1	*27*-*26*-*27*
PBH11	TTCAATCAAGGGACTTATAATTT	3	2	*23*-*37*-*23*-*31*-*23*
PBH12	TTCAATCAAGGGACTTATAATTTTA	3	2	*25*-*35*-*25*-*29*-*25*
PBH13	TAACTTCAATCAAGGCACTTATCACTTTA	4	3	*29*-*31*-*29*-*28*-*29*-*25*-*29*
SKH01	TTCAATCAAGGCACTTATAATTTTA	3	2	*25*-*35*-*25*-*29*-*25*
SKH02	TTCAATCAAGGCACTTATCACTTTA	3	2	*25*-*35*-*25*-*32*-*25*
SKH03	TTCAATCAAGGGACTTACAAATT	4	3	*23*-*37*-*23*-*34*-*23*-*37*-*23*
SKH04	TTCAATCAAGGGACTTACAAATT	4	3	*23*-*37*-23-*34*-*23*-*37*-*23*
SKH05	TTCAATCAAGGGACTTATCACTTTA	4	3	*25*-*35*-*25*-*32*-*25*-*29*-*25*
SKH06	TTTCAATCAAGGGACTTATAACTTTA	4	3	*26*-*34*-*26*-*31*-*26*-*34*-*26*
SKH07	TAACTTCAATCAAGGCACTTATCACTTTA	4	3	*29*-*31*-*29*-*28*-*29*-*25*-*29*

Underline indicates the Consensus sequence

Italic indicates the Direct repeat

Italicunderline indicates the Spacer length

**Fig. 1:**
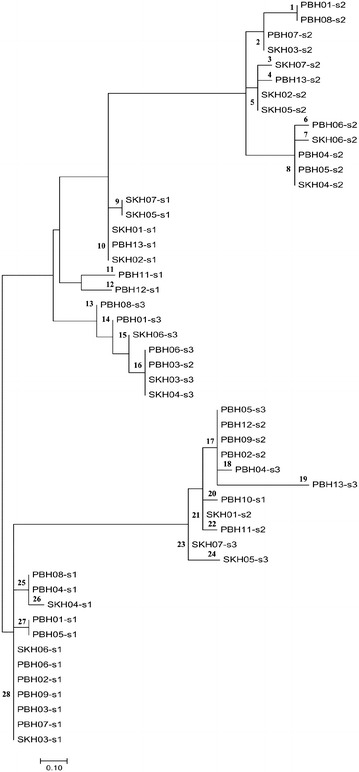
Classification of all spacers obtained in this study based on evolutionary relationships with phylogenetic tree using MEGA7 software. The evolutionary distance scale is 0.1. (PBH01-s2 = spacer no. 2 of *H. pylori* PBH01 isolate)

In this work, a total of 50 spacers were detected. The numbers of spacers in each isolate were between 1 and 3 with lengths from 25 to 37 bp (Table 2). They were classified into 28 different spacer patterns and they were not geographical associated (Fig. 1). Using the CRISPRTarget tool and the NCBI database for evaluation, 18 spacers possessed 83–100% similarity to bacterial plasmid, virus or phage genomes at E value ≤ 0.18 (Additional file 2: Table S1).

### Virulence genes investigation

A total of 36 *H. pylori* isolates was investigated for virulence genes. All of them were positive for *cagA* and *vacA* s1 (Table 3). The most pathogenic genotype (*cagA vacA* s1m1) was detected in 17 out of 29 (58.6%) isolates from PBH and 5 out of 7 (71.4%) from SKH. One isolate from each hospital (PBH 06 and SKH 02) was positive for the *vapD* gene.

**Table 3:** Virulence genes of *H. pylori*

Isolate	Virulence genotype	*vapD* gene
PBH01	*cagAvacA* s1m1	−
PBH02	*cagAvacA* s1m2	−
PBH03	*cagAvacA* s1m2	−
PBH04	*cagAvacA* s1m1	−
PBH05	*cagAvacA* s1m1	−
PBH06	*cagAvacA* s1m1	+
PBH07	*cagAvacA* s1m1	−
PBH08	*cagAvacA* s1m1	−
PBH09	*cagAvacA* s1m1	−
PBH10	*cagAvacA* s1m2	−
PBH11	*cagAvacA* s1m2	−
PBH12	*cagAvacA* s1m1	−
PBH13	*cagAvacA* s1m1	−
PBH14	*cagAvacA* s1m2	−
PBH15	*cagAvacA* s1m1	−
PBH16	*cagAvacA* s1m1	−
PBH17	*cagAvacA* s1m2	−
PBH18	*cagAvacA* s1m1	−
PBH19	*cagAvacA* s1m2	−
PBH20	*cagAvacA* s1m1	−
PBH21	*cagAvacA* s1m2	−
PBH22	*cagAvacA* s1m1	−
PBH23	*cagAvacA* s1m2	−
PBH24	*cagAvacA* s1m1	−
PBH25	*cagAvacA* s1m1	−
PBH26	*cagAvacA* s1m1	−
PBH27	*cagAvacA* s1m2	−
PBH28	*cagAvacA* s1m2	−
PBH29	*cagAvacA* s1m2	−
SKH01	*cagAvacA* s1m1	−
SKH02	*cagAvacA* s1m1	+
SKH03	*cagAvacA* s1m2	−
SKH04	*cagAvacA* s1m1	−
SKH05	*cagAvacA* s1m1	−
SKH06	*cagAvacA* s1m2	−
SKH07	*cagAvacA* s1m1	−

### Genotyping of *H. pylori*

**Fig. 2:**
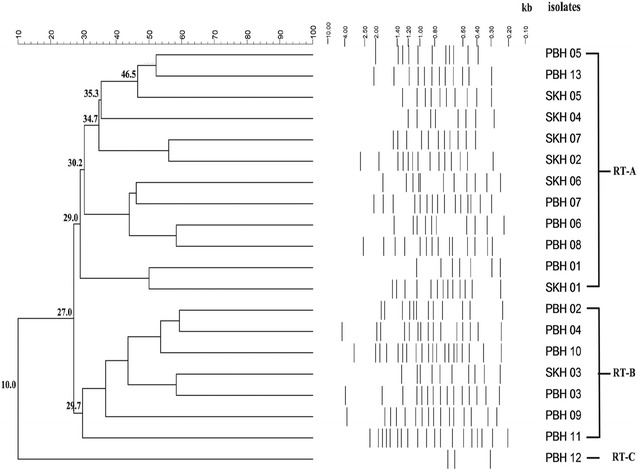
RAPD analysis of 20 *H. pylori* isolates (PBH = 13) and (SKH = 7). Phylogenetic analysis was performed using BioNumerrics 7.6. Similarity (%) between patterns was calculated using the Dice index. The data was sorted using the UPGMA method

Twenty isolates of *H. pylori* were investigated for RAPD and CRISPR-virulence analysis. All of them exhibited different RAPD profiles with fragments between 0.20 and 4.37 kb and could be assigned into three clusters (RT-A, RT-B and RT-C) (Fig. 2). Twelve (60%), seven (35%) and one (5%) isolates were classified in the RT-A, RT-B and RT-C clusters, respectively. Isolates in the RT-A cluster were 73% dissimilar to the isolates in the RT-B cluster whereas the isolate in the RT-C cluster was 90% dissimilar to the isolates in the RT-A and RT-B clusters.

**Fig. 3:**
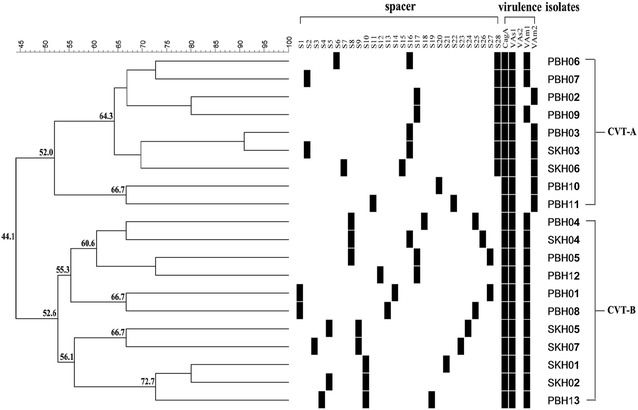
CRISPR-virulence typing of 20 *H. pylori* isolates from PBH (n = 13) and SKH (n = 7) base on binary matrix. Phylogenetic analysis was performed using BioNumerrics 7.6. Similarity (%) between patterns was calculated using the Dice index. The data were sorted using the UPGMA method

CRISPR-virulence analysis revealed 20 different profiles which could be classified into two clusters, CVT-A (9 isolates) and CVT-B (11 isolates). The isolates in the CVT-A cluster were 55.9% dissimilar to the isolates in the CT-B cluster (Fig. 3).

## Discussion

Type II CRISPR-Cas systems are present in *Helicobacter cinaedi* (NC017761), *Helicobacter mustelae* (NC013949) and *Wolinella succinogenes* (NC005090) [28]. In *Helicobacter cetorum* (NC017737) a type III CRISPR-Cas system is detected. However, no *cas* gene could be identified in *H. pylori*, thus CRISPR-like sequences is assigned for the CRISPR loci detected in this work.

**Fig. 4:**
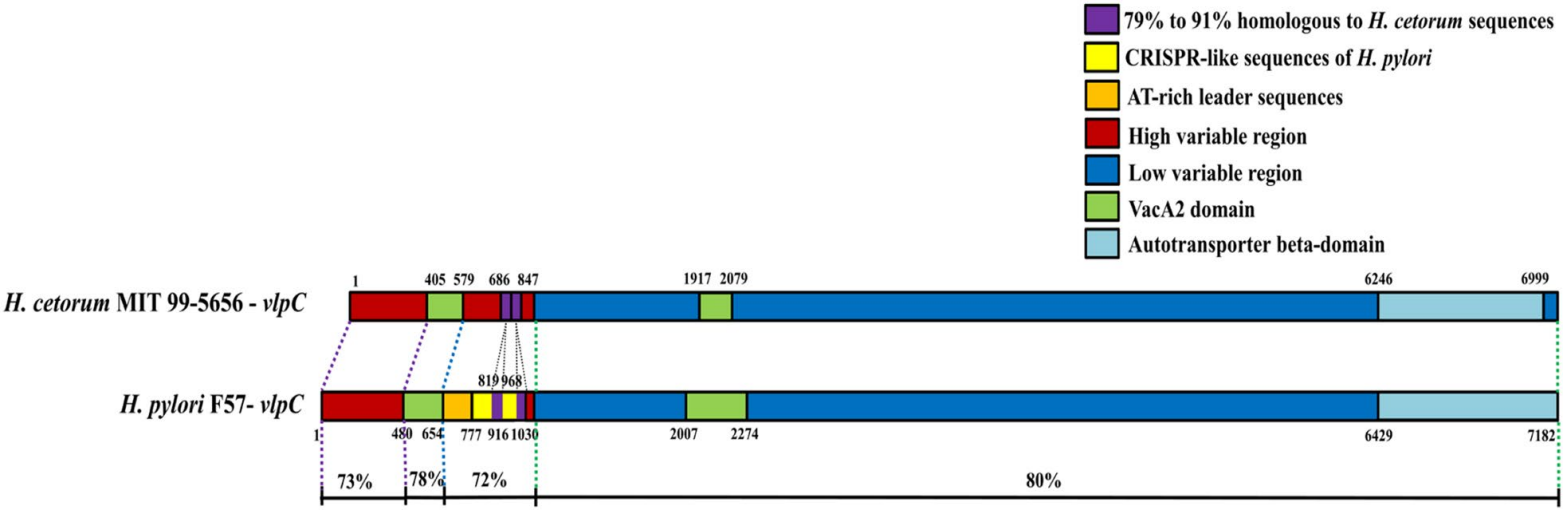
Comparison of *vacA*-like gene (*vlpC*) in *H. pylori* F57 (AP011945.1) and *H. cetorum* MIT 99-5656 (CP003481.1)

It has been reported that *H. pylori* and several *Helicobacter* spp. possess *vacA*-like genes. In *H. pylori*, these genes are designated as *imaA* (immunomodulatory autotransporter A), *faaA* (flagella-associated autotransporter A) and *vlpC* (VacA-like protein C-Vlp C) [29]. It is of interest that all the CRISPR-like sequences of *H. pylori* identified in this work are located in the *vlpC* gene (Fig. 4). Analysis of *faaA* and *imaA* of 5 *H. pylori* isolates (26695-1MET, XZ274, F57, India7, and SNT49) indicated the absence of any CRISPR-like sequences. Although the function of Vlp C has not been clearly illustrated, mutation of the *vlpC* gene is associated with the resistance of *H. pylori* to metronidazole [30]. Analysis of *H. pylori* 26695-1MET Vlp C using the PHYRE2 program (the protein homology/analogy recognition engine v 2.0) indicated that this protein was homologous to the viral A-type inclusion protein (E = 2 × 10^−69^) of the *Trichomonas vaginalis* virus (TVV). Interestingly, the presence of TVV in *T. vaginalis* causes resistance to metronidazole [31]. The NCBI database indicates that only *H. pylori* and *H. cetorum* harbor this gene. Analysis of *vlpC* of *H. pylori* F57 and *H. cetorum* MIT 99-5656 revealed the homology of nucleic sequences to be between 72 and 80% and this gene contained 2 regions, a high variable region and a low variable region (Fig. 4). The CRISPR-like sequences were identified only in *H. pylori* and were located in the high variable region (between 777 and 1030 nucleotide sequences) with AT-rich leader sequences and contained 2 regions (between 819–916 and 968–1030) homologous to *H. cetorum* sequences. AT-rich leader sequences assumed to be a transcriptional promoter are assigned as one of the CRISPR feature structures [22, 32]. Thus, it is possible that the CRISPR-like sequences were inserted into this part of the *vlpC* gene, which is not the conserved domain or functional region of the gene. Analysis of 4210 CRISPR arrays revealed 89% were *cas*-positive in which 33% CRISPR arrays were adjacent to a *cas* locus and 56% were located outside of *cas* loci, the rest 11% were orphans (no *cas* detected) [33]. It has been demonstrated that orphan CRISPR may involve in gene regulation. In *Listeria monocytogenes*, orphan CRISPR affects virulence through *feo*AB iron transport system [34]. In *H. pylori* the presence of CRISPR-like sequences in *vlpC* gene may influence its expression. In vivo investigation of *H. pylori* three *vacA*-like genes revealed that they were up regulated during colonization of mouse stomach; however, in vitro evaluation of VacA-like proteins in broth culture supernatant using Western blot analysis, ImaA and FaaA were detected but Vlp C was not detected which might be due to it was expressed in the low level [35]. The CRISPR-like sequences of *H. pylori* are detected in the coding region of a *vlpC* gene, it is uncommon that CRISPR locus of *H. pylori* located in the coding region, however, it has been reported that CRISPR loci I, III, IV and V of *Thermotaga maritima* were detected in the genes encoded for the hypothetical protein [36]. It is possible that *H. pylori* acquires those sequences after *vlpC* gene is already presented in its genome because no CRISPR-like sequences is detected in the *vlpC* gene of *H. cetorum*.

In this work, we detected 23–31 bp DRs in *H. pylori* and the number of spacers was between 1 and 3, with 25–37 bp. Twenty-eight spacer patterns were classified from *H. pylori* isolates. Interestingly, *H. pylori* designated as PBH03 and SKH03 possessed the same virulence gene pattern (Fig. 3) and s1 and s2 spacers of PBH03 were organized in the spacer pattern 28 and 16 respectively which were the same patterns of s1 and s3 of SKH03 respectively (Fig. 1). Thus, it is possible that those bacterial isolates might be invaded by the same kind of phage. In this work, some spacers were 83–100% similar to genetic elements encoding phage DNA polymerase (PBH03-s2, PBH06-s3, SKH03-s3 and SKH04-s3), phage tail protein (PBH13-s2, SKH02-s2 and SKH05-s2) or phage DNA packing protein (SKH04-s1) whereas some spacers were similar to hypothetical proteins derived from bacterial plasmid (PBH06-s1, SKH03-s1, SKH06-s1) (Additional file 2: Table S1). These genetic elements could be bacterial signatures for typing.

*H. pylori cagA* ^+^, *vacA* s1m1strains have been associated in the development of peptic ulcer and gastric carcinoma more than *H. pylori cagA* ^**−**^, *vacA* s1m2 strains [10, 37]. In this work, all *H. pylori* isolates were *cagA* ^+^ strains and the occurrence rate of *vacA* s1m1 and *vacA* s1m2 strains was 61.1% (22/36) and 38.9% (14/36), respectively. All *vacA* m2 *H. pylori* isolates were organized in the CVT-A cluster of CRISPR-virulence typing (Fig. 3). The previous studies of *H. pylori* in Thailand demonstrated that 95% of isolates were *cagA* ^+^ whereas the *vacA* s1m1 and s1m2 genotypes were found in 65 and 35% of total isolates, respectively [38]. In this study, the prevalence rate of pathogenic *H. pylori* (*cagA* ^+^ *vacA* s1m1) in samples collected in the southern region was 71.4% which was significantly higher than the prevalence rate in the samples from the central region (58.6%).

*H. pylori* VapD protein possesses endoribonuclease activity and may be involved in cell maintenance or the protection of foreign genetic elements [39, 40]. It is of interest that only 5.5% (2/36) of *H. pylori* obtained in this work were positive for the *vapD* gene whereas it was present in 38.0% of *H. pylori* isolates from Mexico and 61.3% from the USA [12, 25]. Using hybridization and confirmation by sequencing, it has been demonstrated that *vapD*-negative *H. pylori* isolates possessed high-level genetic diversity in the *vapD* region [12]. Differences in the detection of this gene in Asian (Thailand) and Western (Mexico and USA) *H. pylori* isolates should be explored.

Various patterns of spacers in the CRISPR-Cas system have been reported for subtyping *C. jejuni*, *Mycobacterium tuberculosis*, *Erwinia amylovora*, *V. parahaemolyticus* and *S. enterica* [8, 20, 41]. Analysis of orphan CRISPR spacers derived from 14 clinical *Enterococcus faecalis* isolates indicates two groups of clonal strains which correlates to the Multilocus sequence typing (MLST) [42]. Better bacterial discrimination through a combination of CRISPR pattern and virulence gene typing has been demonstrated [43, 44]. In the present study, the DI of CRISPR-virulence typing was higher than that of CRISPR typing alone (Additional file 3: Table S2). Analysis of 20 profiles derived from CRISPR-virulence typing and RAPD revealed that DIs of both techniques were 1. However, RAPD seems to be better than CRISPR-virulence typing because it could differentiate *H. pylori* PBH12 from the other strains (Fig. 2) whereas only two clusters were obtained from CRISPR-virulence typing (Fig. 3). This might be due to the low number of spacers detected in the tested isolates in this work. However, the reproducibility of RAPD is poor and the method lacks validated interpretation criteria for inter-laboratory comparison [18, 45]. CRISPR-virulence typing is more reliable for long-term global epidemiology and evolutionary studies and the CRISPR locus databank is excellent for inter-laboratory interpretation and inter-laboratory reproducibility. The information of the CRISPR-like sequences in *H. pylori* acquired in this work expands the basic knowledge for typing of bacteria.

## Conclusions

This is the first report of CRISPR-like sequences in *H. pylori* which was detected in the *vlpC* gene. Twenty-eight different spacers with the numbers between 1 and 3 were identified and can be used as bacterial signature for genotyping. In this work, CRISPR-virulence typing of *H. pylori* was performed and compared with the RAPD. Their DIs were not different. However, RAPD is poor reproducible and lacks validated interpretation criteria for inter-laboratory comparison. Therefore, CRISPR-virulence typing is a novel epidemiological tracking tool for *H. pylori* management.

## Additional files

**Additional file 1: Figure S1.** Clusters of DR assigned in *H. pylori* based on multiplex sequence alignment using MEGA7 software (highlight indicated the share sequences within the cluster).

**Additional file 2: Table S1.** Genetic elements exhibiting similarity to spacer sequences.

**Additional file 3: Table S2.** Discriminatory power (DI) of *H. pylori* genotyping.
