# Correction to: CRISPR-like sequences in *Helicobacter pylori* and application in genotyping

**DOI:** 10.1186/s13099-017-0221-x

**Published:** 2017-12-11

**Authors:** Khotchawan Bangpanwimon, Jaksin Sottisuporn, Pimonsri Mittraparp-arthorn, Warattaya Ueaphatthanaphanich, Attapon Rattanasupar, Christine Pourcel, Varaporn Vuddhakul

**Affiliations:** 1 Department of Microbiology, Faculty of Science, Prince of Songkla University, Hat Yai, Thailand; 2 NKC Institute of Gastroenterology and Hepatology, Songklanagarind Hospital, Faculty of Medicine, Prince of Songkla University, Hat Yai, Thailand; 3 Microbiology Laboratory, Vichaiyut Hospital, Bangkok, Thailand; 4 KC Center of Gastroenterology and Hepatology, Hat Yai Hospital, Hat Yai, Thailand; 5 Institute for Integrative Biology of the Cell (I2BC), CEA, CNRS, Univ. Paris-Sud, Université Paris-Saclay, Gif-sur-Yvette, France

## Correction to: Gut Pathog (2017) 9:65 10.1186/s13099-017-0215-8

In the original version of this article [1], published on 17 November 2017, Table 2 contains an error: the first “T” in the first sequence in the column ‘Consensus direct repeats (CDRs) sequences’ has been incorrectly underlined. In Table 2, the underlining indicates the Consensus sequence.

- The sequence was originally underlined like this:
  AACAGCACTTTCAATCAAGGGACTTACAA
- The sequence should have been underlined like this:
  AACAGCACTTTCAATCAAGGGACTTACAA

The original publication of this article has been corrected.
