# Supplementary material for: CRISPR-like sequences in Helicobacter pylori and application in genotyping
Source: Gut Pathog. 2017 Nov 17;9:65. doi: 10.1186/s13099-017-0215-8 (PMC5693588; doi:10.1186/s13099-017-0215-8)
Supplement: Supplementary file 1 — Additional file 1: Figure S1. Clusters of DR assigned in H. pylori based on multiplex sequence alignment using MEGA7 software (highlight indicated the share sequences within the cluster). [file 13099_2017_215_MOESM1_ESM.doc]

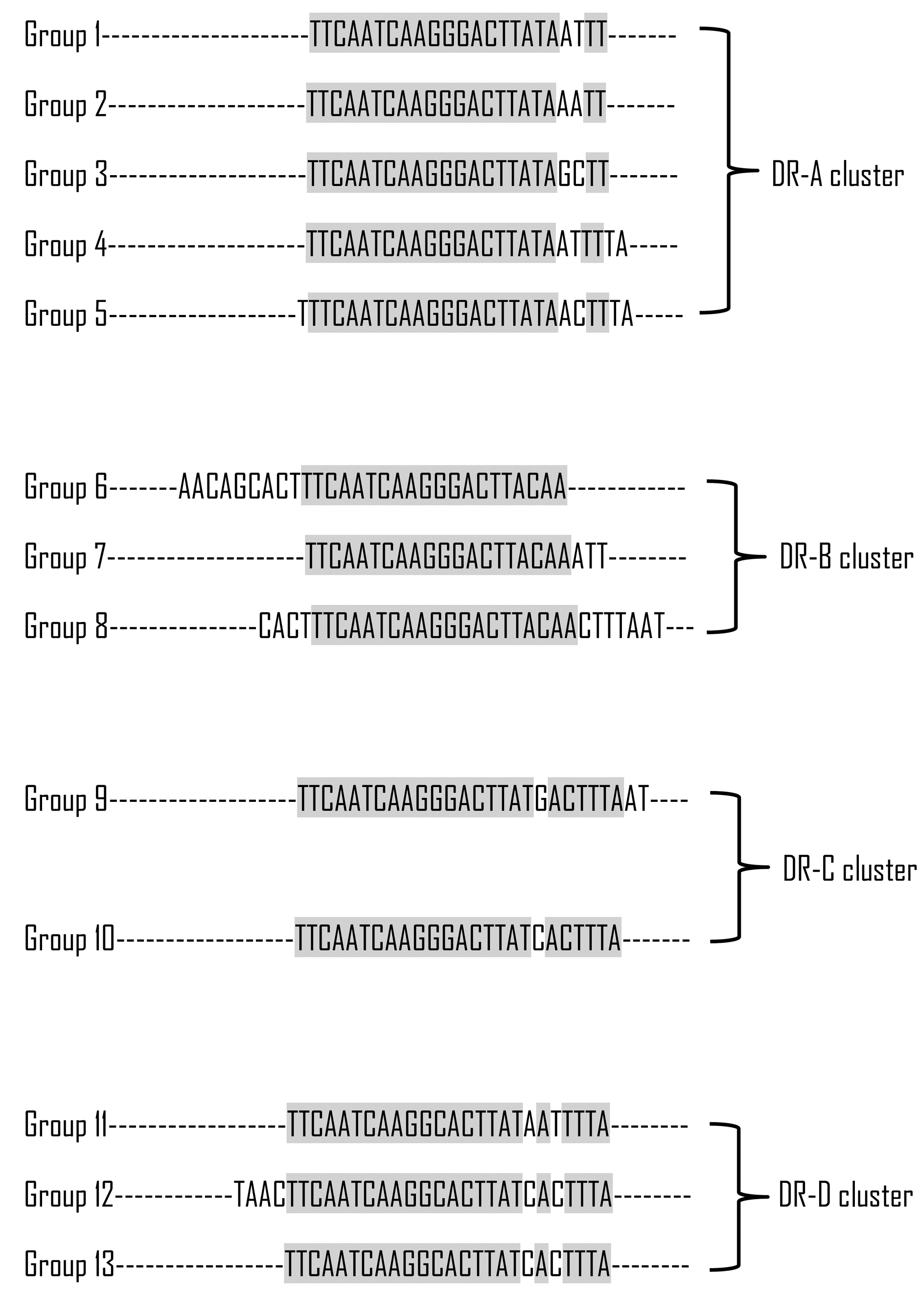


**Fig. S1** Clusters of DR assigned in *H. pylori* based on multiplex sequence alignment using MEGA7 software. (Highlight indicated the share sequences within the cluster)
