# Supplementary material for: CRISPR-like sequences in Helicobacter pylori and application in genotyping
Source: Gut Pathog. 2017 Nov 17;9:65. doi: 10.1186/s13099-017-0215-8 (PMC5693588; doi:10.1186/s13099-017-0215-8)
Supplement: Supplementary file 2 — Additional file 2: Table S1. Genetic elements exhibiting similarity to spacer sequences. [file 13099_2017_215_MOESM2_ESM.doc]

Additional file 2

**Table S1** **Genetic elements exhibiting similarity to spacer sequences**

| Spacers | Sequences of spacer | Similar genetic elements/  hypothetical proteins | Accession Number |
| --- | --- | --- | --- |
| PBH05-s1 | CTAACAGCACTAACTTAAACTTCAACAACAGCGCT | *Prevotella* sp. oral taxon 299 str. F0039 plasmid | NC_022111.1 |
| PBH05-s3 | CTGACAACGCCAGCTTCAATAACGACACC | Weissella phage WCP30 | NC_031101.1, KU981050 |
| PBH06-s1, SKH03-s1, SKH06-s1 | CACTAACAGCGCTAACTTAAACTTCAATAACAGCGCT | *Enterococcus gallinarum* strain FDA_163 plasmid | NZ_CP014068.1 |
| PBH06-s2 | TAATAGCGCTCAATCTGTTTTTAATAACAGCAAT | Equid Herpes Virus 4 | NC_001844.1 |
| PBH06-s3, PBH03-s2, SKH03-s3, SKH04-s3 | TACTGACAATACAGGTTTAAACTTTGATAACGACACC | Clostridium phage phiCTP1 | NC_014457.1, HM159959 |
| PBH13-s2 | ATAGCACCCAATCCACTTTTGAAAACAG | Streptococcus thermophilus bacteriophage Sfi11 | NC_002214.1, AF158600 |
|  |  | Streptococcus phage TP-J34, TP-778L and ALQ13.2 | NC_022776.1, HG380752, NC_013598.1, FJ226752, NC_020197.1, HE861935 |
|  |  | *Klebsiella quasipneumoniae* strain ATCC 700603 plasmid | NZ_CP014697.2 |
|  |  | *Klebsiella oxytoca* strain CAV1374 and KONIH1 plasmid | NZ_CP008789.1, NZ_CP011634.1 |
|  |  | *Klebsiella pneumoniae* subsp. pneumoniae strain KPNIH29 and HS11286 plasmid | NZ_CP009865.1, NC_016846.1 |
|  |  | *Klebsiella pneumonia* strain 628, CAV1042, CAV1193, CAV1344, SWU01, pKPC-LK30 and pKP048 plasmid | NC_032103.1, NZ_CP018670.1, NZ_CP013323.1, NZ_CP011623.1, NZ_CP018455.1, NC_020893.1, NC_014312.1 |
| SKH01-s1 | GTAACAGCGCAAGTTCAAGCTTTGATAATAGTAGT | *Lactobacillus phage Ldl1* | NC_026609.1, KM514685 |
| SKH02-s2, SKH05-s2 | ATAGCGCCCAATCCACTTTTGAAAACAGCAAT | Streptococcus phage TP-778L, TP-J34 and Sfi11 | NC_022776.1, NC_020197.1, NC_002214.1, HG380752, HE861935, , AF158600 |
|  |  | Streptococcus thermophilus bacteriophage O1205 | NC_004303.1, U88974 |
| SKH03-s2 | TAGTAGCGCTCAATCCACTTTTAATAATGATACT | *Borrelia miyamotoi* FR64b plasmid | NZ_CP004223.1 |
|  |  | Lactobacillus phage AQ113 | NC_019782.1, HE956704 |
| SKH04-s1 | CACTAACAGTGCTAATTTAAACTTCAATAACAGTGCT | Enterococcus phage vB_EfaP_IME195 | NC_028693.1, KT932700 |
| SKH06-s2 | GTAGTGCTCAATCTGTTTTTGAAAACAGCAA | *Vibrio tapetis* plasmid pVT 1 | NC_010614.1 |
|  |  | Enterococcus phage vB_EfaS_IME197 | NC_028671.1, KT945994 |
| SKH06-s3 | CTGACAATACAGGTTTAAACTTTAATAACGACAC | Torque teno mini virus 8 | NC_014068.1 |
