# Supplementary material for: CRISPR-like sequences in Helicobacter pylori and application in genotyping
Source: Gut Pathog. 2017 Nov 17;9:65. doi: 10.1186/s13099-017-0215-8 (PMC5693588; doi:10.1186/s13099-017-0215-8)
Supplement: Supplementary file 3 — Additional file 3: Table S2. Discriminatory power (DI) of H. pylori genotyping. [file 13099_2017_215_MOESM3_ESM.doc]

**Table S2 Discriminatory power (DI) of *H. pylori* genotyping**

| Genotyping | DI |
| --- | --- |
| RAPD typing | 1.000 |
| CRISPR typing | 0.995 |
| CRISPR-virulence typing | 1.000 |
